# Boron Toxicity and Deficiency in Agricultural Plants

**DOI:** 10.3390/ijms21041424

**Published:** 2020-02-20

**Authors:** Milka Brdar-Jokanović

**Affiliations:** Institute of Field and Vegetable Crops, National Institute of the Republic of Serbia, Maksima Gorkog 30, 21000 Novi Sad, Serbia; milka.brdar@ifvcns.ns.ac.rs; Tel.: +381-21-780-365

**Keywords:** boron, agriculture, deficiency, toxicity, wheat

## Abstract

Boron is an essential plant micronutrient taken up via the roots mostly in the form of boric acid. Its important role in plant metabolism involves the stabilization of molecules with *cis*-diol groups. The element is involved in the cell wall and membrane structure and functioning; therefore, it participates in numerous ion, metabolite, and hormone transport reactions. Boron has an extremely narrow range between deficiency and toxicity, and inadequate boron supply exhibits a detrimental effect on the yield of agricultural plants. The deficiency problem can be solved by fertilization, whereas soil boron toxicity can be ameliorated using various procedures; however, these approaches are costly and time-consuming, and they often show temporary effects. Plant species, as well as the genotypes within the species, dramatically differ in terms of boron requirements; thus, the available soil boron which is deficient for one crop may exhibit toxic effects on another. The widely documented intraspecies genetic variability regarding boron utilization efficiency and toxicity tolerance, together with the knowledge of the physiology and genetics of boron, should result in the development of efficient and tolerant varieties that may represent a long-term sustainable solution for the problem of inadequate or excess boron supply.

## 1. Introduction

Plants require essential macro and micronutrients for normal growth and development. The inadequate supply of a nutrient, whether leading to deficiency or toxicity, affects plant growth and results in yield and quality losses in agricultural plants. This review deals with boron as a unique chemical element and plant micronutrient.

## 2. Micronutrient Boron

### 2.1. Boron Is an Essential Micronutrient for Higher Plants

Boron (B) is a chemical element belonging to group 13 of the periodic system of elements (III A according to Chemical Abstracts Service (CAS) and III B according to old International Union of Pure and Applied Chemistry (IUPAC) nomenclature), thus displaying metallic and non-metallic properties, with the capability of producing both acids and bases. It is taken up by plant roots predominantly in the form of small uncharged boric acid molecules which easily enter the cell by passing through the wall’s phospholipid bilayer [1,2,3]. Boron is a plant micronutrient, i.e., it is necessary for normal growth and development in amounts as small as 1 μ/g–1 mg/g.

Since the mid-19th century it is known that plant organisms contain boron [4]. Its presence was scientifically proven in several plant species including wheat [5], although without claims of the element being essential. Boron was studied as a micronutrient by Mazé in 1915 [6]; however, the experimental techniques used by the author were later questioned. Therefore, Warington [7] is considered to be the first author proving the necessity of the element for plant metabolism. It was later confirmed that boron is an essential micronutrient for several monocotyledonous and dicotyledonous species [8], conifers [9], ferns [10], and phytoplankton species [11]. Soon, the importance of the element for agricultural plants in general was recognized [12]. Boron is an essential or at least highly beneficial micronutrient for animal organisms, affecting the metabolism of macro minerals Ca, P, and Mg, triglyceride, glucose, amino acids, proteins, reactive oxygen species, and steroid hormones. For humans, the recommended daily intake is 1–13 mg, and the primary sources are fruits, vegetables, nuts, and legumes [13,14,15,16,17].

### 2.2. Roles of Boron in Plant Metabolism

It is known for almost a century that boron is an essential micronutrient; however, its role in plant metabolism is still being debated [18,19]. The primary function of the element is in cell-wall synthesis, and in maintaining its structure and integrity. The amount of boron located in the wall depends on the plant species, organ, and boron supply [20]. Plants take up boron in the form of small uncharged boric acid molecules (about 96%), as well as borate anions to a significantly lesser extent [21]. Both compounds easily form complexes with compounds having *cis*-hydroxyl groups. Borate forms ester bonds between apiose residues of rhamnogalacturonan II (RGII) monomers, thus contributing to the cell wall’s architecture and function. Generally, monocotyledonous plants contain less pectin in their cell walls than dicotyledonous plants; therefore, they have comparatively lower boron requirements and lower tolerance to excess boron [22,23,24,25,26,27,28,29,30,31,32,33,34,35,36].

It was hypothesized [21] that boron’s primary role in plant metabolism may be the stabilization of molecules with *cis*-diol groups, no matter which function they have. Boron forms *cis*-diol complex compounds with glycoproteins and glycolipids from the plasma membrane, thus maintaining its structure. It plays a role in membrane metabolism and function, thereby being involved in enzyme reactions, as well as the transport of ions, metabolites, and hormones [2,37,38,39]. The element stimulates, inhibits, or stabilize enzymes; it is involved in the transport of sugars across the membrane, lignin and flavonoid synthesis, and metabolism of auxins, nitrogen compounds, and phenols [32,40,41,42,43,44].

Root growth cessation observed in conditions of insufficient boron supply results from the inhibition of DNA synthesis, as a result of pyrimidine base deficits [38]. The element is involved in cell division [41,45], tissue vegetative growth and differentiation [46], translation and transcription [47], and nitrogen fixation in legumes and mycorrhiza [21,48].

Boron deficiency affects the photosynthetic capacity and the transport of photosynthetic products [39,49,50,51,52]. A decrease in stomatal conductance and CO_2_ assimilation, and an increase in intercellular CO_2_ concentrations were found in boron-deficient *Citrus sinensis* leaves. Hexose and starch accumulation is increased and sucrose accumulation is decreased, implying feedback regulation in CO_2_ assimilation. Antioxidant enzymes remain active; however, the system as a whole is not sufficiently effective in protection from oxidative damage [53]. In order to maintain net carbon balance under deficiency, respiration, organic-acid metabolism, amino-acid biosynthesis, and the anapleurotic pathway are upregulated in leaves while downregulated in roots. The phenolic concentration is increased in both leaves and roots [54,55].

Inadequate boron supply causes numerous biochemical, physiological, and anatomic changes; therefore, it is extremely difficult to distinguish with certainty the primary and secondary effects of the deprivation. Interruption of cell division in the apical meristem is the most prominent effect that results in a reduction and even cessation of root growth. Boron deficiency decreases male fertility by reducing microsporogenesis, germination, and elongation of the pollen tube [56]. After fertilization, the nutrition disorder affects embryogenesis, resulting in seed deterioration or the formation of incomplete or damaged embryos [30,31,41,57,58,59].

### 2.3. Boron Uptake

Boron uptake was considered a controversial topic for a long time, inter alia due to various plant species, extremely high boron concentrations, and different techniques used in the experiments [60]. Plants take up boron via the roots, predominantly in the form of boric acid. It is a small, soluble, undissociated, and uncharged molecule which easily migrates across the lipid bilayers. Boron is the only element which is not taken up from the soil as an ion. In conditions of sufficient supply, the element is transported by passive diffusion and without protein catalysis and energy consumption. Because of the cell’s high permeability to boron, characteristic patterns of flux along the transpiration stream, and accumulation in the tips of the leaves, passive diffusion was long considered as the only mechanism of transport [1,61,62]. Linear increases in boron tissue content following the increase of external boron [63], as well as the fact that metabolic inhibitors and both low and high temperatures (2–42 °C) do not impair the accumulation [64], seemed to confirm this point of view. It was suggested that the element enters the cell partially via passive diffusion through the lipid bilayer, and partially via protein channels, aquaporins, or other channels that are Hg-sensitive [2].

The substantial differences among plant species regarding boron mobility make the element unique among nutrients. Boron is rapidly and significantly phloem-mobile in species for which polyols (e.g., sorbitol, mannitol, dulcitol) are the primary products of photosynthesis. The mobility is due to boron’s complexation with polyols. Such species are from *Prunus*, *Malus*, and *Pyrus* genera (prune, pear, apple, cherry, almond, plum, peach, apricot), as well as onion, celery, carrot, olive, bean, pea, cauliflower, cabbage, asparagus, and coffee. On the other hand, boron has restricted phloem mobility in species with sucrose as a primary photosynthetic product. These species (e.g., wheat, barley, walnut) represent the majority. Boron moves along the transpiration stream and accumulates at its end. Therefore, the concentration of the element is dramatically higher in leaf tips and margins than in the rest of the leaf. On the contrary, boron concentration is uniform across the leaves of phloem-mobile species. Consistently, comparatively higher boron concentration in older leaves implies restricted phloem mobility. A higher concentration in young leaves and fruits indicates significant phloem mobility [65,66,67,68].

The dramatic inter- and intraspecies differences in plant tissue boron content, as well as in boron tolerance and sensitivity, undermined the passive transport hypothesis. Plant tissue boron content was generally considered to be lower in tolerant species and genotypes [33,62,69,70,71], although exceptions were reported [72,73,74,75]. Nevertheless, differences in tolerance cannot be explained exclusively by differences in transpiration, because the above would mean that tolerant plants have to exhibit seven-fold higher water use efficiency than sensitive plants [76]. Higher cell-wall pectin content [27,29], inactivation in the wall or cytoplasm [76,77,78], separation into the vacuole [79,80] and cell wall [81], redistribution among plant organs [82], blocking the entrance in tolerant genotypes via decreased permeability of root cell plasma membranes [62,69,83], and efflux from the roots [84,85] were hypothesized as possible mechanisms for plant boron tolerance. Nevertheless, active transport has to be involved in the tolerance [86,87,88].

Because passive diffusion is not capable of satisfying plant boron demands under deficiency, boric acid channels and borate exporters have to be involved in uptake and translocation to growing tissues. Exporters are additionally involved in exclusion in conditions of excessive boron. The channels transporting boric acid are of the major intrinsic protein (MIP) family, subfamily nodulin 26-like intrinsic proteins (NIPs). The first such channel was identified in *Arabidopsis* and named NIP5;1 [89]. It functions in root cells exposed to boron deprivation and it is downregulated if external boron increases [90,91,92]. NIP6;1 is involved in boron transfer from the xylem to phloem [93] and NIP7;1 in microsporogenesis [94]. Homologous channels were found in barley, maize, and rice [95,96,97,98].

Borate transporters (BOR) functioning under boron deficiency were firstly determined in *Arabidopsis* [93,99,100,101], and its homologues were reported for rice, wheat, maize, and other plant species [102,103,104,105,106]. Seven BORs in *Arabidopsis* were identified so far. BOR1 is involved in boron transport from roots to the xylem and its translocation to leaves, collaborating with NIP5;1 and NIP6;1, respectively. BOR2 supports root growth by participating in crosslinking RGII molecules [107,108], whereas BOR4 functions primarily in tolerance to excessive boron via exclusion from tissues. BOR4 homologues were found in barley, wheat, and other agricultural plants [85,109,110,111,112]. In addition to the boron availability in the medium, external factors such as CO_2_ and irradiance affect the function of these proteins [113].

However, the elucidation of the mechanisms via which plants cope with boron stresses remains a difficult task. For example, the proteins involved in the response to boron deficiency in citrus roots belong to functional groups related to cell transport, biological regulation and signal transduction, stress responses, and other (protein, nucleic acid, carbohydrate and energy, cell wall and cytoskeleton, lipid) metabolic processes. The root adaptation to low boron might include up- and downregulation of microRNA involved in decreased respiration, improved ability to scavenge reactive oxygen species, enhanced cell transport, increased lateral root number, improved osmoprotection, and other metabolic reactions [114,115]. In addition, there are many differences between roots and leaves in adaptive mechanisms to low boron at the transcriptional level; most of the differentially expressed fragments related to signal transduction and stress defense are down- and upregulated in roots and leaves, respectively [116]. Several genes involved in cell-wall metabolism and transmembrane transport are highly regulated under boron deficiency in citrus. Numerous metabolic pathways (lignin biosynthesis, nitrogen metabolism, and glycolytic pathway) are affected by stress [117]. The advanced knowledge on the physiological basis of boron uptake and distribution should facilitate the breeding of plants tolerant to boron deficiency and excess. Genes with transcript accumulations induced by boron deficiency were identified in several studies [89,118,119], as well as the first identification of a transcription factor gene induced by low boron (WRKY6), involved in normal *Arabidopsis* root growth under deficiency [120].

## 3. Boron in the Environment

### 3.1. Sources of Boron

Although present in small amounts, boron is widely distributed in the hydrosphere and lithosphere. It enters an environment naturally or through anthropogenic processes. The most common sources of naturally occurring soil boron are borosilicate mineral tourmaline, volatile volcano emanations, geothermal streams, groundwater, and seawater. Tourmaline is stable and accumulates in sedimentary rocks. The boron from the silicate is unavailable to plants before weathering in the pedosphere. After the degradation process, the element is accessible to plants in the form of boric acid. Global seawater boron concentrations are estimated as approximately 4.6 mg∙L^−1^. The element is often found in saline soils originating from marine evaporites.

Boron is commercially derived from the mineral ulexite, borax (tincal), natural boric acid (sassolite), colemanite, and kernite, and the richest sources of this element are located in the United States (US) and Turkey. The element is used in the manufacture of fiberglass, thermo-stable borosilicate glass (Pyrex) and porcelain, detergents, enamels, synthetic herbicides, and fertilizers, as well as in metallurgy for nuclear shields. The use of a number of boron compounds in electronics, as well as for the production of aviation and rocket propellants, is common. In this regard, anthropogenic influences on boron releases into the environment are predominately via irrigation water, although the element often enters the ecosystem as wastewater, fertilizer, herbicide, combustion product, and waste from mining or processing industry. There are agricultural plants (sugar beet, carrot, alfalfa) that tolerate high boron concentration in irrigation water, up to 4 mg∙L^−1^. When grown on soils with high adsorption capacity, even sensitive crops irrigated with boron-rich water can give satisfactory yields [76,121,122,123,124,125]. However, prolonged irrigation with such water may have a detrimental effect on soil. The threshold for boron in irrigation water that does not harm soil sustainability is 1 mg∙L^−1^ [126].

Boric acid is a soluble compound easily leached by rainfall; thus, boron deficiency commonly occurs in humid areas. Areas in southwestern China, northwestern India, Nepal, Japan, Bangladesh, and Brazil are considered as the most endangered by boron deficiency; however, the disorder may be expected in at least 80 countries in the world, including Zambia, Nigeria, Philippines, Thailand, Koreas, north European countries, and the Balkans [59,127,128]. In contrast to boron deficiency, soil boron toxicity is less abundant and occurs in arid and semi-arid areas. Excessive soil boron was reported in Australia, US, Russia, Turkey, Mexico, Israel, Iraq, Egypt, Syria, Morocco, Jordan, Libya, India, Pakistan, Malaysia, Peru, Chile, Hungary, Serbia, and Italy [76,129,130,131,132].

### 3.2. Soil Boron

It is a difficult task to determine limit values for micronutrients in soil, which is especially true for boron. The majority of the world’s agricultural soils contain 5–30 ppm total boron, determined by total digestion of air-dried soil at neutral pH. The maximum amount of the element that should not affect crops is 25 ppm [133]. However, only a small fraction of the total soil boron (1–3%) is hydrosoluble and, therefore, available to plants as a nutrient. Other fractions (specifically adsorbed, oxide and organically bound, residual B) interact and can mutually transform under specific environmental conditions [50,134,135].

The range between boron deficiency and toxicity is extremely narrow, narrower than for any other element [136]. Generally, soils with less than 0.5 ppm hot-water-extractable boron are considered deficient, while only a few ppm may result in toxicity [72]. According to Allison [137], 0.7 ppm is optimal while concentrations higher than 1.5 ppm represent toxicity for sensitive plant species. Abreu et al. [138] summarized their research with findings of other authors [139,140] and proposed the following scale: 0.0–0.2 ppm as low, 0.21–0.6 ppm medium, 0.61–1.1 ppm high, 1.2–3.0 ppm very high, and >3.0 ppm as toxic soil boron concentrations. It was suggested [141] that 0.5–2.0 ppm represents the optimal soil boron range, whereas lower and higher values indicate deficiency and toxicity. Disorders in boron nutrition are, therefore, quite common. Depending on the circumstances, both deficiency and toxicity may occur at the same locality [142], even during the same growing season [143]. In addition, critical levels depend on soil type, pH, water status, texture, air humidity and temperature, plant species, and genotype [127,144]. For example, it was found that boron concentrations exhibiting toxic effects on barley (root growth reduction by 10%) varied about 10-fold among 22 tested soils differing in pH, texture, and percentage of organic matter [145].

Average hot-water-extractable soil boron concentrations that are considered to be deficient are 0.19 ppm in Nepal, 0.25 ppm in Zambia, 0.27 ppm in Nigeria, 0.28 ppm in Philippines, 0.37 ppm in Korea, 0.39 ppm in Sierra Leone, and 0.42 ppm in India. Toxic concentrations of 0.68, 1.02, 1.10, 1.26, and 1.51 ppm were measured in Pakistan, Hungary, Turkey, Mexico, and Iraq, respectively [127].

Boron deficiency in wheat was noticed and recognized 50–60 years ago, during the “green revolution”, i.e., the expansion of semi-dwarf varieties in eastern Nepal, northeastern India, northwestern Bangladesh, and southwestern China [58]. The deficiency was found in more than 130 plant species and occurred in almost all countries of the world [127,146,147]. However, it was not until 1984 that boron toxicity was recognized in cereal crops. A yield reduction of 17% noted in barley (cultivar Clipper) grown in the Australian cereal belt was found to be due to high soil boron. The barley shoot and grain boron concentrations were 96.0 and 6.6 ppm, respectively. The characteristic brown necrotic spots on the leaves that indicate toxicity were previously attributed to fungal (*Pyrenohora teres* f. ssp. *maculata*) infestation [148]. Although the toxicity predominately occurs in warm arid (<250 mm) and semi-arid regions (250–450 mm annual precipitation) [76,149], it can be manifested in cold areas with low humidity [73]. In this case, plants uptake the same amount of the element which they would adopt in optimal temperature and water regimes. However, the root growth is reduced following the cold dry winter. Consequently, the visual symptoms of boron toxicity occur in the early phases of the plants’ life cycle.

The effect of excess soil boron on crops depends on its vertical distribution. For example, in most Australian soils, the concentration of boron increases from the soil surface to a depth of approximately 1 m, where it reaches the maximum [150,151]. Such a distribution of the element in field conditions may delay the visual symptom appearance to later plant growth stages. In drought, the roots penetrate deeper into the soil, facing excessive boron, which results in more pronounced symptoms of the toxicity. In experiments conducted under controlled conditions, excessive boron is commonly added during seeding, which strengthens its effects compared to field conditions [151,152]. In this respect, plants are not equally sensitive to excess boron at all growth stages. Barley is particularly sensitive during stem elongation [153]. Moreover, plants with toxicity symptoms are often irregularly distributed in the field. Barley plants with and without visual symptoms were found in the field up to 10 m apart. The plants had shoot boron concentrations of 32 and 5 ppm [150,151].

In spite of the widely documented linear relationship between external boron and the concentration of the element in the plant tissue ([69] pot experiment, [62] nutrient solution, [151] field conditions), there is great intraspecies variability in boron tolerance. For example, wheat genotypes without yield reduction at 100 ppm soil boron treatment were identified; however, genotypes with significant yield reduction at 25 ppm boron treatment are also known [69]. Plant and soil boron concentrations are not necessarily directly correlated. Barley plants with shoot boron concentrations of 323 and 156 ppm were sampled at localities with soil boron of 21.6 and 56.3 ppm, respectively [150]. There was no correlation between the severity of the plant symptoms and soil boron concentration in another field study [154]. Similarly, soil boron deficiency is not necessarily correlated to the deficiency in plants [58].

Substantial research was done in the attempt to quantify the fraction of soil boron that is available to plants. This is a difficult task due to the physical and chemical properties varying among soils, the effects of environmental conditions, and the differences in boron uptake among plant species and varieties within the species. The first developed and most commonly used method was the hot-water-soluble method [155]. The procedure is difficult to standardize due to the effects of extraction temperature and time, as well as the potential boron resorption during the cooling [156]. Therefore, other methods were developed, such as those using hot dilute calcium chloride [157], cold calcium chloride–mannitol [158], cold hydrochloric acid [159], barium chloride and microwave-heated water [160], diethylene triamine pentaacetic acid (DTPA)–sorbitol [161], and potassium chloride extraction [162]. The results are substantially in agreement with those obtained via the standard hot water extraction method. The quantity of the extracted boron is further determined using spectrophotometric and plasma-source spectrometric methods. The validation of newly developed promising soil tests [141,163] in a wide range of soils, environmental conditions, and crops should contribute to a better understanding of complicated plant–soil interactions concerning boron uptake, translocation, and utilization.

### 3.3. Alleviation of the Effects of Boron Deficiency and Toxicity

The deficiency of boron is generally managed through the application of appropriate boron fertilizers. The most commonly used practice is soil fertilization, although foliar application and seed priming can be applied as well. The effects of the deficiency on plants can be more or less successfully alleviated, depending on the method of choice, time of application, other soil characteristics, temperature and humidity, and the species and genotype efficiency of boron uptake and utilization. The performance of numerous agricultural plants was improved using boron fertilizers, most importantly in terms of yield, but also in terms of quality and certain physiological parameters, e.g., water management and chlorophyll content. Given the narrow range between deficiency and toxicity, the dosage of the added boron should be carefully adjusted for each application [164,165,166,167,168,169,170,171].

Amelioration of the soils containing excess boron can be performed by leaching, adding various amendments, or growing tolerant plants. Leaching with boron-deficient water, e.g., river water, is applied following the successful reclamation of saline soils. The procedure has to be adjusted to physical and chemical characteristics of the soil. The impediments regarding boron leaching are a lower rate of removal in comparison to salt, possible migration in deeper soil layers and consequential contamination of the root zone, the risks of leaching other nutrients, and the risk of boron regeneration from natural reserves [76,172,173]. The relationships between boron and other elements, such as Ca, S, Zn, Si, and Al were used for developing amendments that are intended either for soil improvements or for the alleviation of the toxic effects on plants [174,175,176]. Organic matter, as well as various plant growth modulators, was applied in attempts to mitigate excess boron [177,178]. Developing and applying cost-effective methods for boron removal from irrigation water would solve the problem of the most common anthropogenic source of contamination [124,179,180,181].

## 4. Agricultural Plants and Boron

### 4.1. Boron Deficiency

Root growth is more sensitive to boron deficiency than shoot growth. The cessation of cell division in the apical meristem resulting from boron deficiency inhibits root elongation. Under severe deficiency, the root cap disappears, the growth stops, and root tips die out. The shoot/root ratio increases, and plants become more susceptible to drought and nutritive imbalances. Longitudinal splits close to the primary vein of young leaves and leaf margins deformed in shape were described in wheat by Snowball and Robson [182]; however, these symptoms are rarely seen in the field [58]. In the case of severe and prolonged deficiency, the internodes are shortened, and necrotic spots appear on leaves.

Boron requirements are generally higher during the reproductive phase of a plant’s life cycle. Consequently, in field conditions, the yield can be significantly reduced without the appearance of visual symptoms of the deficiency [30]. The critical level for deficiency in the vegetative phase is 1 ppm in flag leaves of wheat; however, similar to toxicity, leaf boron concentration is not necessarily a reflection of boron utilization efficiency [183,184]. On the other hand, 7–8 ppm in anthers and 5–6 ppm in carpels result in wheat sterility [185,186]. The most common effect of deficiency during the reproductive phase is male sterility. If fertilization does occur, seeds may be aborted. Accordingly, the most pronounced effect of boron deficiency in, e.g., wheat is a reduced number of seeds per spike [187] and, in extreme cases, yield can be 100% reduced due to nutrition disorder [188]. Genotypes with stable yield in the conditions that reduce yield in other genotypes are regarded as having more efficient boron utilization [189].

Phloem-mobile species rarely experience deficiency, thanks to their ability to retranslocate internal boron. Therefore, the increased production of polyols may be an effective strategy for improving boron utilization efficiency. This may be achieved through transformation (e.g., tobacco, rice), or via the selection of genotypes with high polyol production [33,66,190,191].

There is a wide variation among wheat genotypes in terms of boron utilization efficiency, and it is probably the widest variation of all agricultural plants and nutrients. Therefore, breeding for utilization efficiency was proposed as the most feasible way to overcome the deficiency problem [59,109,165,184,185,187,188,192,193,194,195,196]. It was found that boron efficiency in wheat is controlled by two independent major loci. Dominant loci (Bod1, Bod2) imply high efficiency; thus, it is believed that the standards for utilization efficiency feature the following combinations of loci: Bod1 Bod1, Bod2 Bod2 (Fang 60—efficient cultivar), bod1bod1 bod2bod2 (Bonza—inefficient), and bod1bod1 Bod2 Bod2 (SW 41—medium efficient), according to Jamjod et al. [197].

The utilization efficiency is conditioned by a high capacity for boron transport from roots to aboveground plant organs [198,199]. Takano et al. [101] carried out the first identification of the *Arabidopsis thaliana* membrane protein BOR1 responsible for loading boron in the xylem and its translocation in conditions of deficiency. The protein is homologous to the bicarbonate transporter in animals. A generation of *Arabidopsis* that is tolerant to boron deficiency was produced [109]. This was achieved via increased BOR1 production causing an increased boron translocation from roots to aboveground organs. The transgenic plants successfully yielded in deficiency conditions that reduced wild-type yield by 100%, and they did not exhibit increased sensitivity to excess boron. The transporters that were later identified in major agricultural plants represent the basis for further research aimed at breeding or designing varieties with improved boron utilization efficiency [35,105,200,201].

For example, transgenic tomato lines (cultivar Micro-Tom) with improved growth in boron deficiency conditions were designed [202]. The transformation was *Agrobacterium*-mediated. Tomato is often used as a model of fruit-bearing crops. The lines are boron-efficient due to a strong expression of *AtBOR1* (*Arabidopsis thaliana* BOR1). When compared to non-transgenic plants, transgenic plants grown in deficiency conditions had higher shoot dry weight, as well as shoot and fruit boron concentration. The results of this study imply that transgenic crops may be a sustainable solution for boron-deficient areas. Another approach may be marker-assisted selection breeding for boron utilization efficiency. The trait is polygenic in most agricultural plants. In *Brassica napus*, e.g., efficiency includes boron uptake, transport, and utilization. Thanks to the previously constructed high-density genetic map, several quantitative trait loci (QTL) involved in boron utilization efficiency were identified. The development of molecular markers associated with the QTLs should help breeders to identify efficient genotypes. However, substantial research remains to be done to transfer the knowledge on the physiology and genetics of boron utilization efficiency into boron-efficient plant varieties with good growth, quality, and yield [201,203,204,205,206].

### 4.2. Boron Toxicity

Symptoms of boron toxicity differ between species with restricted and significant phloem mobility. In phloem-immobile species, boron moves via the xylem and accumulates at the end of the transpiration stream. Accordingly, foliar symptoms in barley and wheat include chlorosis and necrosis spreading from the leaf tips, with brown lesions that are at first formed on the margins, and then cover much of the leaf surface. The oldest leaves are first affected by the disorder, which further spreads to the top of the plant. In severe cases, brown lesions are present at leaf sheaths, stems, spikes, and awns. Delayed emergence and delayed foliation, as well as a reduction in stem height, dry matter weight, 1000-kernel weight, number of spikes per plant, and yield, were reported [62,69,71,73,130,136,148,207,208,209]. Generally, symptoms are more pronounced and earlier exhibited in barley than in wheat [72]. If boron concentration increases with soil depth, symptoms are manifested later, and yield is less reduced [151]. Excessive boron does not affect leaf area, width, or length [152]. General root weakness and decreased growth of lateral roots were described in hydroponically grown wheat and barley [62]. The severity of all boron toxicity symptoms varies among genotypes, e.g., there are those with lower, the same, or higher dry matter weight in boron treatments [74]. Reduced yield, as well as significant variability among genotypes in terms of both yield reduction and visual symptoms of boron toxicity, was observed in wheat and barley [69,148,152,209,210,211]. Furthermore, there are genotypes with stable yields in conditions of elevated soil boron, despite pronounced toxicity symptoms and high shoot boron concentration [154].

In maize, tomato, carrot, and alfalfa, excess boron decreases emergence [208]. Young shoot tip cessation, leaf axil gumming, brown corky lesions along stems and petioles, and bud abscission were reported for phloem-mobile *Prunus*, *Malus*, and *Pyrus* species. In celery, irregular stem shape and deformed young leaves occur as symptoms of boron toxicity [66].

Dramatical differences in literature data concerning plant boron concentrations may be explained by plant species (boron mobility), organ (e.g., concentration in wheat flag leaf is about 10-fold higher than in grains [212]), stage of life (older plants contain more boron [213]), treatment boron concentration, genotype, and experimental technique [147,214,215]. Generally, in wheat, 68% of plant boron is located in the leaves, 16% in roots, 10% in spikes, and 6% in stems [196]. Field trials are under the influence of numerous environmental factors; however, the experimental design of the trials performed in controlled conditions also affects boron uptake. Pot size and type, temperature, watering regime, and the intensity of the applied treatments are among the factors influencing nutrient uptake in controlled conditions [216]. Plants take up a significantly higher quantity of boron in the laboratory than in the open field [209]. Critical barley shoot boron concentration was 30 ppm when grown in the field, while several genotypes grown in glasshouses achieved maximum yield at 140 ppm [150]. Critical plant boron concentration is that which results in more than 10% reduction of the yield [151].

The lowest critical shoot boron concentrations suggested for wheat and barley are 10–16 ppm [181,217]; however, in another study, they were in the range of 10–130 ppm [76]. In the trials performed in controlled conditions, the critical shoot boron concentrations were 60 ppm [218] and 80 ppm [62,219]. In another experiment [147], wheat genotypes with critical boron concentrations of both 44 and 318 ppm were identified. The range of 324–648 ppm was determined in the shoots of durum wheat treated with excessive boron [74]. In the shoots of the field-grown plants, toxic boron concentrations were from 10–30 ppm in wheat and barley [218], to even 68–323 ppm in barley [150]. According to the results of another study [151], critical boron concentrations in barley shoots were 4–76 ppm, and those in the flag leaf were 273 ppm.

As a species with restricted phloem mobility, barley has dramatically higher (10–50-fold) boron content in leaf tips compared to the leaf base, suggesting that leaf concentration depends on the proportion of the tip and the remaining leaf parts. Boron accumulation is influenced by transpiration; in conditions of increased water use, boron accumulation and its concentration in the leaf tips increase [63]. Excessive external boron reduces water transport and transpiration in *Arabidopsis*, which could act as a mechanism of boron tolerance [220]. Moreover, boron is easily leached from the leaves by rain [77,78]. Therefore, universal critical boron concentrations are almost impossible to define.

The generally accepted opinion is that tolerant barley and wheat genotypes have lower boron concentration in all organs, regardless of the type of the experiment and soil or nutrient solution boron concentrations [33,69,70,71,221]. However, variability in shoot boron concentration of barley, bread, and durum wheat genotypes tolerant to excess boron was reported [72]. Therefore, some genotypes take up or translocate less boron, and some genotypes tolerate higher tissue boron concentration. For example, 70 durum wheat genotypes were examined, and tolerant and sensitive genotypes with high and low shoot boron concentrations, respectively, were identified [74]. In a laboratory trial that included 40 bread wheat genotypes screened for boron tolerance at a seedling stage, no relationship was found between parameters of tolerance and plant boron concentration [75]. On the other hand, the tolerant genotypes had comparatively higher boron content which was explained by reduced uptake of all nutrients including boron in the sensitive genotypes.

Evidently, the type of the experiment, treatment strength, boron measurement units, genotype, growth stage, and plant organ affect optimal and critical boron concentration in agricultural plants.

Several authors attempted to investigate a possible connection between the geographical origin of genotypes and boron tolerance. Wheat cultivars originating from India, Japan, and Afghanistan are mostly tolerant [213], similar to barley and durum wheats from Iran, Afghanistan, Syria, Pakistan, and Turkey [130,222]. However, in the group of bread wheat genotypes from Argentina, Turkey, and Iraq, the authors recorded significant variation in terms of boron tolerance. The greatest variability was identified in Italian varieties. Wheat genotypes from Australia, US, Canada, Egypt, and northwest Europe proved to be susceptible [213], as well as the Russian [72] and European barley genotypes [223]. Maize, carrot, tomato, and alfalfa germplasm originating from Chile proved to be more tolerant to boron than the North American germplasm [208].

Among the genotypes of various origins, the difference in B tolerance might be explained by an edaphic adaptation, i.e., most often unintended selection toward boron tolerance in breeding centers located in the regions with an excess soil boron problem. Several examples confirm this point of view. Growth and yield in several genotypes of 42 agricultural plants in the trial set in northern Chile were examined [222]. Agricultural land in the region was irrigated with saline water containing high boron for centuries. Local varieties yielded significantly better than expected in such conditions. Boron tolerance was investigated in field-grown wheats of different origin [224,225], and local cultivars (Turkey and Serbia, respectively) were distinguished as the most adapted to the nutrition disorder. Two Syrian local populations were tested, one commonly grown in a semi-arid region prone to boron toxicity and another from the region with higher precipitation. As expected, the first was tolerant and the second susceptible to excess boron [223].

Out of 45 Australian wheat genotypes tested for utilization efficiency, 44 were inefficient [186]. Similarly, the International Maize and Wheat Improvement Center (CIMMYT) germplasm is generally inefficient, especially durum wheat. Out of 1108 lines of barley, triticale, bread, and durum wheat, about three-quarters are susceptible to boron deficiency [189]. However, in spite of the toxicity problem in Australia, the majority of wheats are susceptible to excessive boron. This was explained by the narrow genetic variability of the starting material used in breeding. The medium-tolerant cultivar Federation is often in the pedigree of Australian cultivars [213].

The first research aimed at investigating the genetics of boron tolerance in wheat was performed by Paull research group [70,149,209], when at least three unlinked major genes acting in an additive manner were identified. The loci were located on chromosomes 4AL, 7BL, and 7DL. The 7B locus (Bo1) was extensively investigated as it represents the source of boron tolerance in the majority of Australian wheat varieties [84,95,111,226,227,228,229].

Considering the narrow range between boron toxicity and deficiency, the costs and constraints related to boron fertilization and amelioration of boron-excessive soils, the effects of environmental conditions on its uptake and utilization, the identified vast intraspecies variability in terms of the utilization efficiency and tolerance, and the recent findings related to its physiology and genetics, the best solution which may be possible to accomplish in the future would be to breed varieties of agricultural plants that are adjusted to the wide range of soil boron concentrations.

## 5. Summary

Boron is in many ways unique among plant nutrients; however, it is especially distinguished by the substantial differences among species in terms of mobility, the narrow range between deficiency and toxicity, and differential inter- and intraspecies response to an inadequate supply. Both boron deficiency and toxicity may have detrimental effects on yield of various agricultural plants. In addition to fertilization, growing varieties that are efficient in boron utilization were proposed to solve problems related to boron deficiency. The effects of this nutrition disorder are more pronounced in the reproductive phase of plant life, and in species in which the element is phloem-immobile. Both transgenic and marker-assisted selection breeding approaches may be effective strategies to improve crops’ boron utilization efficiency. Unlike deficiency, soil boron toxicity is much more difficult to ameliorate; therefore, growing tolerant crops may be the only sustainable solution. Vast intraspecies genetic variability, together with novel findings on the mechanisms of boron toxicity tolerance, should facilitate breeding varieties with satisfactory yields in boron excessive soils.

## Abbreviations

B	Boron
CAS	Chemical Abstracts Service
IUPAC	International Union of Pure and Applied Chemistry
RGII	rhamnogalacturonan II
MIP	major intrinsic protein
NIP	nodulin 26-like intrinsic protein
BOR	borate transporters
DTPA	diethylene triamine pentaacetic acid
QTL	quantitative trait locus
CIMMYT	International Maize and Wheat Improvement Center
